# Potential Mitochondrial Toxicants: Tox21 Screen Identifies Structures of Interest

**DOI:** 10.1289/ehp.123-A23

**Published:** 2015-01-01

**Authors:** Carol Potera

**Affiliations:** Carol Potera, based in Montana, also writes for *Microbe*, *Genetic Engineering News*, and the *American Journal of Nursing*.

Mitochondria have many important functions, including production of adenosine triphosphate (ATP) to fuel cells and regulation of cell growth, signaling, differentiation, and apoptosis.^1^ Disrupted mitochondrial function raises the potential for health effects and in fact has been associated with cancer, diabetes, cardiovascular disease, and autism.^2^^,^^3^ In a study reported this month in *EHP*, investigators with the Tox21 consortium assessed the impact of more than 8,300 chemicals on mitochondrial activity.^4^

Tox21, short for Toxicology in the 21st Century, is a collaboration of federal entities including the National Toxicology Program, the Environmental Protection Agency, the Food and Drug Administration, and the National Center for Advancing Translational Sciences (NCATS). One product of Tox21 to date is a library of more than 10,000 chemical samples comprising 8,312 unique substances. These samples include industrial chemicals, consumer products, food additives, and human and veterinary drugs.^5^

**Figure:**
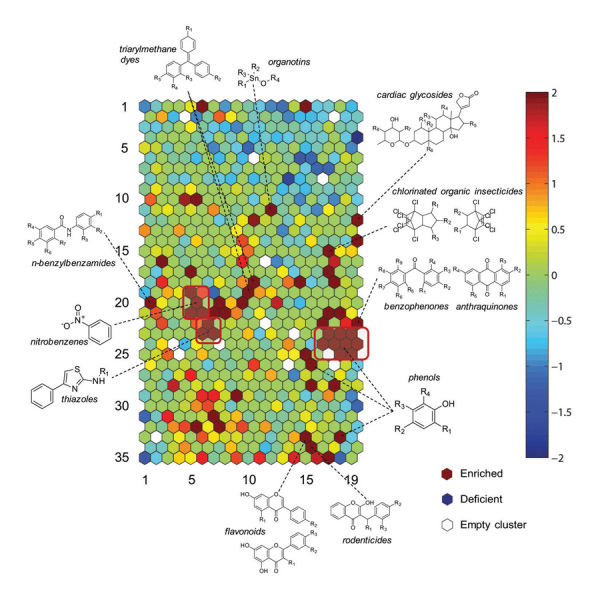
Test chemicals from the Tox21 library were arranged in 651 clusters based on structural similarity. Compared with the library overall, “enriched” clusters showed above-average evidence of harming mitochondria, reflected as a decrease in MMP. Identifying structural features associated with decreased MMP allows researchers to choose appropriate candidate chemicals for further research. Source: Attene-Ramos et al. (2015)^4^

The Tox21 robotic system loads chemical samples and cells into 1,536-well microtiter plates; then the cells are scanned for specific changes.^6^ For the current study, researchers measured mitochondrial membrane potential (MMP) and intracellular ATP content in human hepatocellular carcinoma cells exposed to all 8,312 chemicals. Decreased MMP likely indicates harm to mitochondrial structure and activity, making this end point a good first step for flagging chemicals that interfere with mitochondrial bioenergetics. Changes to intracellular ATP content serve as a marker for both mitochondrial function and cell viability.^4^

Of the chemicals tested, 11% decreased MMP (but with no apparent effect on cell viability), 65% were inactive, and 3% increased MMP. The remaining 21% of the chemicals gave inconclusive results for a variety of reasons. Of these, 17% showed evidence of cytotoxicity.^4^

Some results paralleled previously identified mechanisms of toxicity for chemicals. For example, triethyltin bromide, which is already known to interrupt ATP production,^7^ also proved to be one of the most potent suppressors of MMP.^4^

Structure–activity relationship analysis gave clues about how other chemical may reduce MMP. Recurring structural features associated with decreased MMP included a substituted phenol moiety, a nitrobenzene core, and a thiazole substructure.^4^ “The cell model employed casts a broader net for defining mitochondrial toxicity to include targets beyond the electron transport chain,” says Kendall Wallace, a professor in the Department of Biomedical Sciences at the University of Minnesota, who was not involved with the study.

“We found several clusters not previously known to decrease MMP,” says senior author Menghang Xia, leader of the Systems Toxicology laboratory for the Tox21 program at NCATS. These included parabens with aliphatic side chains of varying lengths. “Their potency correlates with the length of the aliphatic side chain,” says Xia, suggesting these chemical structural features may predict mitochondrial damage. However, experimental research will be necessary to confirm this.

Craig Beeson calls Xia’s work “a tour de force that will go a long way to helping us better understand why so many different chemical classes exhibit mitochondrial damage.” The discovery that potency corresponded to small structural perturbations such as alkyl chain length in parabens is particularly exciting, says Beeson, an associate professor of drug discovery and biomedical sciences at the Medical University of South Carolina, who was not involved with the study.

Xia’s research team also evaluated the performance of the multiplex, high-throughput screening assay itself. “To decrease false-negative and false-positive rates, each chemical was tested in triplicate runs,” Xia explains. “We want to be certain that an inactive compound is accurately defined as inactive, and an active compound truly is active.” All the compounds were tested at 15 different concentrations.^4^

When comparing the multiple dose–response curves generated for each chemical, the mismatch rate was only 0.55% for MMP and 0.03% for ATP.^4^ “Our data quality from the primary screening was very high and reliable,” Xia says.

Now the team is further analyzing the chemicals that decreased MMP but did not change ATP content. Xia says these chemicals will be screened in rat and human hepatocytes to measure MMP changes. Some will also be tested in *Caenorhabditis elegans* (roundworms) and mitochondrial gene microarrays. The combined results will help guide the selection of compounds for in-depth animal studies.
